# Additively Manufactured 316L Stainless Steel Subjected to a Duplex Peening-PVD Coating Treatment

**DOI:** 10.3390/ma16020663

**Published:** 2023-01-10

**Authors:** Luana Bonnici, Joseph Buhagiar, Glenn Cassar, Kelsey Ann Vella, Jian Chen, Xiyu Zhang, Zhiquan Huang, Ann Zammit

**Affiliations:** 1 Department of Metallurgy and Materials Engineering, University of Malta, MSD 2080 Msida, Malta; 2 School of Materials Science and Engineering, SEU University, Nanjing 211189, China

**Keywords:** additive manufacturing, shot peening, PVD multi-layer coating, mechanical testing, corrosion, residual compressive stresses

## Abstract

This research studies the individual and combined effects of mechanical shot peening and the deposition of TiAlCuN coating on additively manufactured 316L stainless steel. Shot peening has been found to induce a 40% increase in surface hardness, while the combined effect of shot peening and the coating produced an approximately three-fold increase in surface hardness when compared to the as-printed coupons. Shot peening reduced the surface roughness of printed metal coupons by 50%, showing that shot peening can also serve to improve the surface finish of as-printed 316L stainless steel components. The peening process was found to induce a compressive residual stress of 589 MPa, with a maximum affected depth of approximately 200 μm. Scratch testing of the printed and coated specimens showed complete delamination failure at a normal load of 14 N, when compared to hybrid treated samples which failed at 10 N. On the other hand, from the corrosion tests, it was found that the hybrid treated samples provided the optimal results as opposed to the other variables.

## 1. Introduction

The marine transportation industry makes up part of the global economy and trade, since primary necessities such as petroleum, foods and goods are carried by means of water. Due to their working environment, parts such as propellers and shafts are exposed to the adverse effects of corrosion, erosion, wear, and detrimental effects. The failure of the fundamental components of a ship can lead to severe consequences, mainly delays in delivery of cargo, environmental damage, financial losses and most importantly fatalities of the passengers on board [1]. Such mid-journey failure would dictate a potentially long sourcing processing and docking of the vessel. The high demand for replacement of parts could be sustained by additive manufacturing (AM).

Components suitable for the marine environment necessitate a particular combination of attributes to make them suitable for use, first and foremost hardness. Additive manufactured 316L stainless steel (SS) is known to have a higher hardness than wrought 316L SS. In a study by Yusuf et al. [1], an average microhardness value of 228 HV was obtained for AM 316L SS, while 192 HV was measured for wrought 316L SS [1]. This increase in microhardness is attributed to the finer grains within the microstructure [2]. Additionally, parts intended for engine and drive components need to possess a high tensile and yield strength to be able to withstand the high loads experienced during use. AM can produce a wide range of ultimate tensile strengths ranging from 550 to 700 MPa, while values for the yield strength vary from 300 to 600 MPa, depending on the chosen parameters used for printing the material [3,4,5,6]. Similarly, corrosion resistance is also an important characteristic for marine transportation parts due to the environment they are subjected to. Few studies on the corrosion resistance of additively manufactured 316L SS are available and no broad consensus has been reached in terms of AM performance compared to wrought equivalents. The corrosion resistance of 316L SS can be enhanced with its fine sub-granular microstructure. This is because a more uniform passive film is generated with the help of the rapid interface boundary diffusion process [7], while other work has shown that SLM components exhibited inferior corrosion resistance in comparison to wrought 316L SS, due to retained porosity [8,9,10]. Jung et al. [8] studied the corrosion characteristics electrochemically in seawater, where AM 316L SS emerged to have poorer qualities than wrought 316L SS.

The typically high surface roughness and residual porosity of AM parts can also cause a deterioration in the mechanical strength of critical components, weakening their performance. The application of surface treatments such as shot peening (SP) and coating deposition, is proposed to mitigate such issues. The cold work induced at the surface via SP can be beneficial in minimising pore diameter found in additively manufactured components, while also inducing work hardening, causing surface texturing and mitigation of intergranular corrosion. In addition, the characteristic resultant residual compressive stresses offset any remnant tensile stresses originating from the printing process [11]. In a study carried out by Santa-aho et al. [12], surface compressive stresses reaching up to 502 MPa were generated in the additively manufactured 316L SS, up to a depth of 80 µm. The compressive stresses generated in the AM 316L SS were quickly formed as the shots impacted the surface, removing the tensile stresses generated by the printing process [12], thereby enhancing fatigue performance [13,14]. Concurrently, shot peening produces an increase in the hardness of the surface and near-surface layers of the material [15,16,17,18]. Gundgire et al. [16] obtained a hardness of 340 HV after SP additively manufactured 316L SS with a native hardness of 230 HV. A similar result was obtained by Sugavaneswaran et al. [15], where the hardness was increased from 230.8 HV to 324.5 HV after SP additively manufactured 316L SS. Furthermore, SP is known to reduce the mean surface roughness (R_a_) of additively manufactured 316L SS. Evidence from research by Rautio et al. [14] shows that after SP additively manufactured 316L SS using martensitic chromium shots, the R_a_ was reduced from 8.81 ± 0.06 μm to 3.93 ± 0.01 μm. Additionally, in another study by Gundgire et al. [16], it was noted that the R_a_ was reduced to 5.81 from 8.81 μm, after SP.

According to the author’s knowledge, literature on coatings deposited by PVD on AM 316L SS is scarce—even more so in the case of novel coating systems such as TiAlCuN. Coatings deposited by PVD are mainly intended to increase the hardness of the substrate on which they have been deposited, as they have a hardness ranging between 2300 and 4079 HV [19,20], while specific additives can be used to provide specific characteristics depending on the intended application. In this case, Cu was added to the more common Ti–Al–N system for anti-biofouling purposes. Furthermore, the coating deposition enables the enhancement of tribological behaviour and corrosion resistance amongst others. Multi-layer coatings are known to have a high corrosion resistance when compared to monolayer coatings and the bare substrate [21,22,23]. This is consistent with work by Ananthakumar et al. [23], a positive shift from −0.837 V to −0.546 V in the corrosion potential was detected when comparing the bare substrate to the multi-layered coated substrate. On top of this, PVD coatings do not typically increase the roughness of the substrate as these conform readily with the underlying surface, making them beneficial as they do not alter the surface once deposited [24]. The coating provides corrosion protection and improvement of tribological behaviour, amongst others. In a study carried out by Tillmann et al. [25], improved adhesion was discovered after the coating deposition of CrAlN coatings on additively manufactured 316L SS.

The combination of SP and coating deposition by PVD is required to increase the hardness, to induce residual compressive stresses and to increase the corrosion resistance of the substrate. Multiple studies have been carried out on the separate application of SP and PVD on AM parts. However, according to the authors’ knowledge, there is a lack of studies on the duplex combination of such surface treatments, with particular attention to those performed on AM-ed 316L SS. Therefore, the aim of this research was to study in detail, the combined effect of shot peening and PVD on AM 316L SS, mainly through the characterisation of microstructure, hardness, roughness, phase analysis, residual stress measurement, the resultant adhesion of the coating to the substrate and the corrosion resistance in a marine simulated environment.

## 2. Materials and Methods

### 2.1. Powder Processing and Manufacturing of Coupons

316L SS powder used for additive manufacturing (AM), having the composition found in Table 1(a), was provided by Jiangsu Vilory Advanced Materials Technology Company Limited (Xuzhou, China). Its particle diameter ranged from 15 to 53 μm [26]. AM was carried out by an AM Pro SP100 3D printer selective laser melting (SLM) machine (Suzhou, China) having a IPG 2000 W type laser, a laser power of 200 W and a laser speed of 950 mm/s. The beam was offset for 10 μm and each layer was 30 μm thick. Samples were printed along the y-direction and had a 20 mm diameter and a 7 mm height. Wrought 316L SS (Table 1(b)) was used as a benchmark, thereby eliminating morphological variation caused by the AM process.

### 2.2. Tensile and Impact Testing

**Figure 1:**
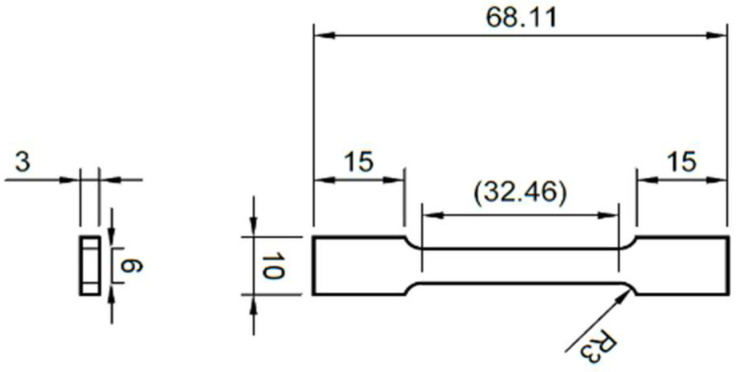
Schematic diagram for the tensile testing specimen. (Units are in mm).

Tensile and impact tests were carried out to study the bulk tensile strength and toughness of the material. The tensile samples having dimensions shown in Figure 1 were printed according to the standard ASTM E8/E8M-16a: Standard for Tension Testing of Metallic Materials [28]. The sub-sized dimensions were chosen so that the samples could be printed within the printer’s limited build volume. The tensile tests were carried out along the x-direction using an Instron Universal Testing System 5892 (Norwood, MA, USA) with the attachment of an Instron extensometer 2530 (USA). A strain rate of 1 mm per minute was used until the elastic region was exceeded and was then changed to 3 mm per minute after removing the extensometer, which strain rate was kept until fracture occurred. Six repeated tests were carried out.

**Figure 2:**
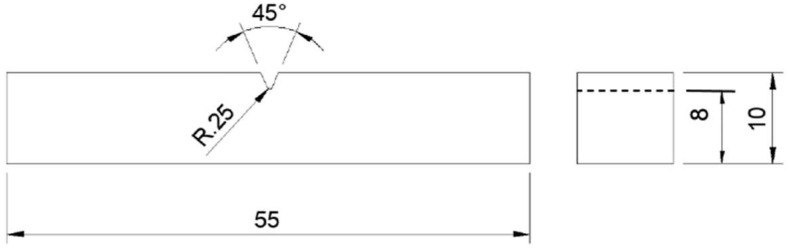
Schematic diagram for the impact testing specimen. (Units are in mm).

The Charpy impact testing samples having dimensions of 55 × 10 × 10 mm, were manufactured according to the standard ASTM E23-18: Standard Test Methods for Notched Bar Impact Testing of Metallic Materials [29], as shown in Figure 2. The impact testing was performed using an Instron 450MPX-J2 (USA) motorised pendulum impact testing system and a velocity of 5.32 m/s. The reported results are the average of six measurements.

### 2.3. Shot Peening and Coating Deposition

Shot peening was carried out using a modified set up in an Industrial Surface Treatments Ltd. AB850 air blasting machine and S230 shots. A nozzle of 80 mm length and 7 mm diameter, nozzle to specimen distance of 100 mm, pressure of 7 bar and an Almen intensity of 0.21 mmA were used.

The coating deposition was carried out using a Teer UDP800 (Beijing, China) closed field unbalanced magnetron sputtering ion plating machine. The system was composed of 2 titanium targets, 1 aluminium and 1 copper target, with an argon and nitrogen gas inlet. Table 2 shows a summary of the coating deposition parameters.

### 2.4. Material Characterisation

Scanning electron microscopy (SEM) was performed by a Carl Zeiss Merlin field emission scanning electron microscope (Oberkochen, Germany) having a Gemini II column. Electron dispersive spectroscopy (EDS) was performed by means of an Ametek EDAX Apollo X 2189 (Mahwah, NJ, USA) attachment located within the SEM. This was used to obtain the chemical composition and elemental maps of the studied surfaces. X-ray diffraction (XRD) phase analysis of all the specimens was carried out using a Rigaku Ultima IV X-ray diffractometer (Tokyo, Japan), equipped with crossbeam optics (CBO) set in Glancing Angle Incidence Asymmetric Bragg (GIAB) configuration, with a 3° angle of incidence and a scan range between 20° and 120°.

Residual stress measurement at the surface and near the surface up to a depth of 200 μm was carried out using the XRD, according to the standard BS EN 15305 (2008)—Non-destructive testing—Test method for residual stress analysis by X-ray diffraction [30], using the sin^2^ψ method. The measurement for peak shifting for the calculation of the residual stresses was performed on the (311) austenite peak at a 2θ value of 90.68°. Each surface was tilted at seven different ψ angles between 0 and 60°. The θ-2θ scans were performed between 2θ values of 87° and 93°. An electrolyte consisting of 5.4% perchloric acid, 94% ethanol and 0.6% de-ionised water was used to carry out electropolishing on a Struers LectroPol-5 electrolytic polishing machine (Copenhagen, Denmark) to progressively remove layers of the material.

Surface micro-hardness tests were executed on a Mitutoyo MVK-H2 micro-hardness testing machine (Kawasaki, Japan), equipped with a Vickers pyramidal indenter and loaded with a 200 gf indentation load, having a dwell time of 10 s. For coating evaluation, nano-indentation tests were performed on a mirror-finished coated wrought 316L SS sample. A Nanomaterial NanoTest 600 machine (Wrexham, UK) was equipped with a 18580-a Berkovich 120° diamond tip indenter. The maximum indentation load was set to 50 mN. Thirty indentations were made on the specimen, spaced at 30 μm from each other.

The surface roughness was measured using an AEP Technology NanoMap 500 LS 3D contact profilometer (California, USA), equipped with a 1 μm stylus tip. The results presented are an average of five measurements.

### 2.5. Scratch Testing

A UMT Bruker TribolabTM tribometer (San Jose, CA, USA) having a 60° Rockwell type C indenter was used to execute micro-scratch testing. A ramped load starting from 0.5 N up to 40 N was used, with a force of 1.33 N/s and a scanning velocity of 0.339 mm/s. Five scratches of 10 mm each were done on the sample. The scratch morphology was then studied under the optical microscope and SEM to identify the positions and loads at which failure took place.

### 2.6. Corrosion Studies

Cyclic polarisation tests were performed on the cylindrical samples using a three-electrode setup to study the corrosion response of the material. The three-electrode setup was connected to a Gamry Interface 1000^TM^ potentiostat (Warminster, PA, USA), having the sample as the working electrode, a platinum coated titanium rod as the counter electrode and a saturated calomel electrode (SCE) as the reference electrode. Then, 300 mL of testing solution was prepared according to ASTM D 1141–98: Standard Practice for the Preparation of Substitute Ocean Water [31], where 1 cm^2^ of surface area was exposed to the electrolyte. An initial OCP test of 2 h was performed, followed by cyclic polarisation sweeps at a rate of 0.167 mV/s, which was reversed at an apex current density of 0.5 mA/cm^2^. Three repeats were performed.

### 2.7. Designation of Samples

Table 3 shows the samples which were tested in this study, together with respective abbreviations used throughout this article.

### 2.8. Error Calculation

The data presented in this work are the sample mean ($\bar{x}$) value obtained from the measured values, with a sample size (n), specified in each section. The quantitative data presented in graphical formats have been included with error bars, while that presented in numerical formats has been included with a ±value. This was done to ensure the correct statistical interpretation of the data. Since most of the sample sizes were smaller than 30, the error ranges were calculated using the t-distribution [32].

## 3. Results and Discussion

### 3.1. Mechanical Performance of Bulk AM 316L SS

#### 3.1.1. Tensile Tests

Tensile properties including the Young’s Modulus, yield strength, ultimate tensile strength (UTS) and elongation are recorded in Table 4.

The measured Young’s Modulus, the yield strength, UTS and the elongation all fall within the ranges found in the available literature for additive manufactured 316L SS. While the UTS of the AM samples is similar to that found in literature for wrought 316L SS, some discrepancies are evident for Young’s Modulus, yield strength and elongation values. A 19% decrease in Young’s Modulus is evident between AM SLM and conventionally manufactured 316L SS. Similar values were obtained by Merkt [41] with a Young’s Modulus of 140 GPa on AM 316L SS. This could be attributed to the parameters of the 3D printing process, specifically the build-up direction and laser power used, which provide different crystallographic orientation of the grains [33,42]. Niendorf et al. [42] report that the grains have a preferential crystallographic orientation according to the laser power utilised such that the grains were oriented in the (011) direction when a laser power of 400 W was used, while the grains were oriented in the (001) direction with a laser power of 1000 W [43]. In austenitic SS, the preferential orientation is that of (001), giving a decreased Young’s Modulus [42]. In addition, the porosity present in additive manufactured materials also results in a decrease in the Young’s Modulus. An increase of 60% in the yield strength, 4% in UTS and 10% in elongation can be noted for AM SLM over wrought. The significant improvement in YS can be attributed to refined microstructure obtained during the high cooling rates of the SLM process. Additionally, the high yield strength and elongation are attributed to high dislocation densities and twinning formations during the SLM process, respectively. Tensile stresses in SLM materials also lead to a high yield strength, with the high dislocation densities formed during deformation [36,44]. The small increase of UTS indicates that during testing, SLM materials do not exhibit the same amount of work hardening as the wrought.

#### 3.1.2. Impact Tests

A total impact energy, resulting in impact toughness, of 75 ± 2 J was obtained (Table 5) after fracturing the sample (Figure 2). This value falls within the range found in literature for AM SLM 316L SS. This value is slightly lower than that of wrought 316L stainless steel.

**Figure 3:**
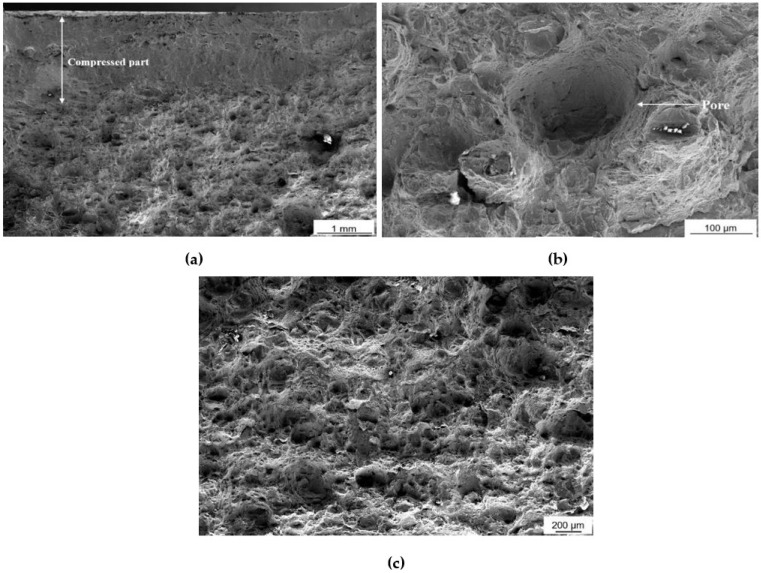
Images portraying the fractured surface of the notched sample (**a**) Compressed part after fracture, showing brittle behaviour. (**b**,**c**) Characteristics of ductile behaviour.

A micrographic analysis of the fractured surface was carried out, as shown in Figure 3a–c. The material which has experienced compressive loading during impact testing shows a distinctively flattened morphology, whereby the material texture both that formed by plastic deformation and features characteristic of AM were compressed against each other, as shown in Figure 3a. In fact, cross-sectional evaluation has revealed some residual porosity of less than 0.2% in volume. Ductile deformation is evidenced by the cup-and-cone structure shown in Figure 3c and pores (Figure 3b), cracks and dimples. The pores, a characteristic of ductile fracture, are formed due to insufficient bonding of melt pools which are next to each other, during the solidification processing [49,50].

### 3.2. Surface and Microstructure Analysis

**Figure 4:**
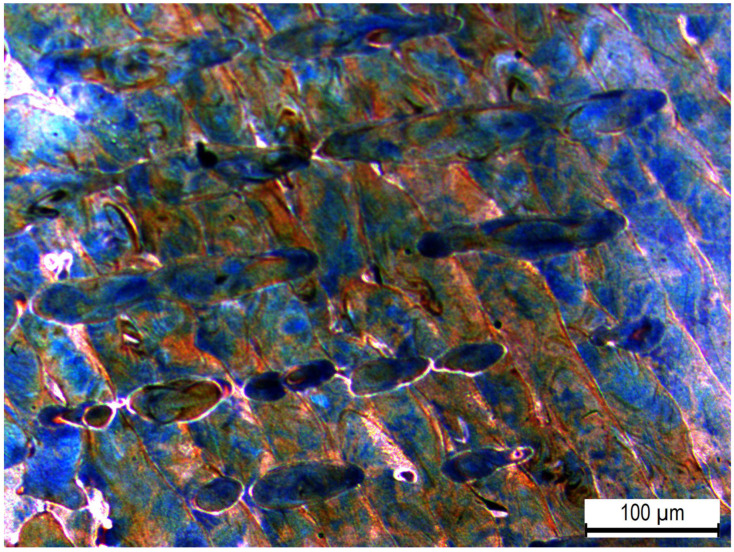
Austenitic microstructure of as-printed 316L SS.

**Figure 5:**
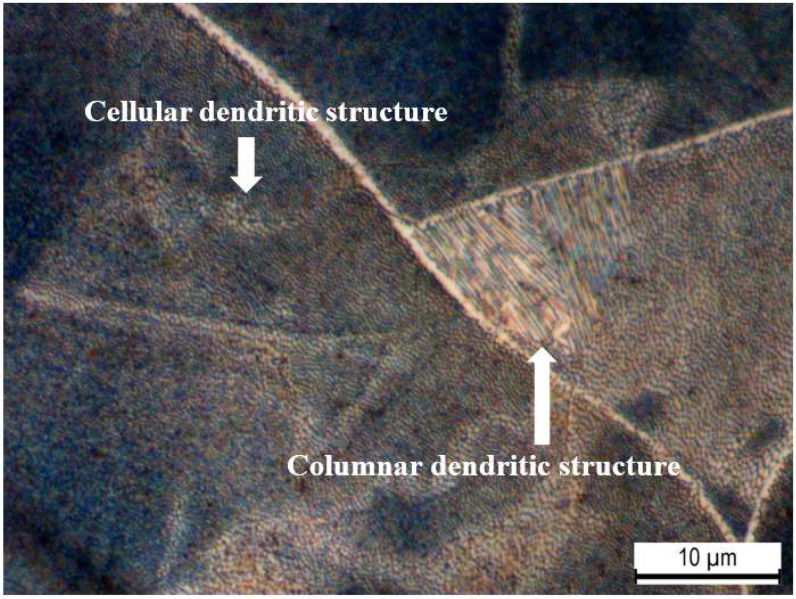
Columnar and cellular dendritic structures in the AP microstructure.

**Figure 6:**
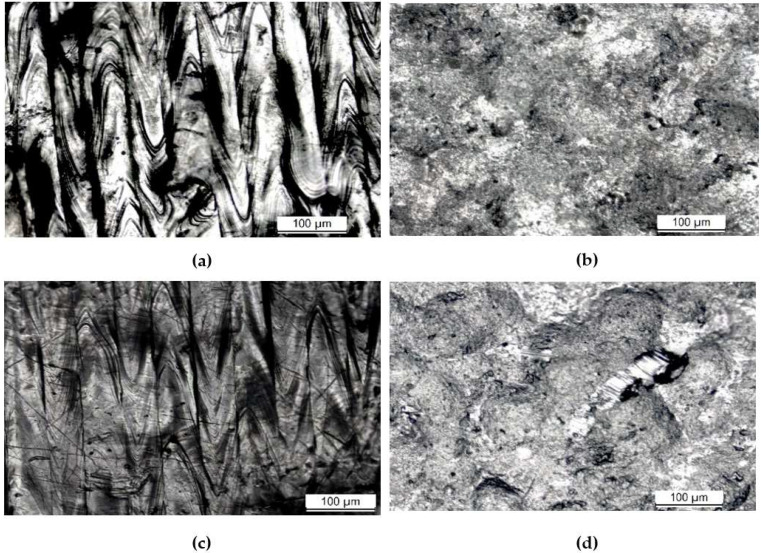
Micrographs for the surface: (**a**) AP (**b**) PSP (**c**) PC and (**d**) PSPC.

Figure 4 shows the microstructure of the AP 316L SS composed of an austenitic matrix. At higher magnifications, shown in Figure 5, columnar and cellular dendritic structures were observed. Such structures are formed following molten metal solidification. Additionally, Figure 6a–d respectively show optical micrographs of the surface of AP, PSP, PC and PSPC specimens. These micrographs show the 100% coverage produced by the shot peening. Figure 6b,d show the individual SP dimple characteristics. SP generated a less rough surface than the as-printed, as will be observed later in Section 3.3.

### 3.3. Roughness Analysis

**Figure 7:**
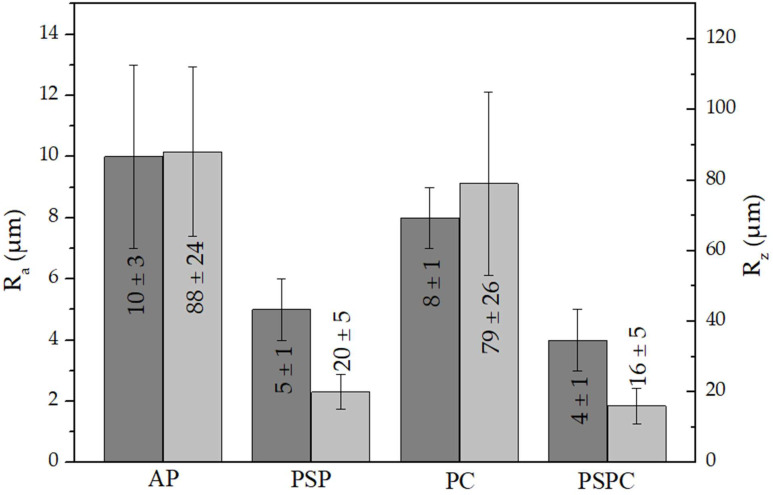
R_a_ and R_z_ roughness values for as-printed, printed and shot peened, printed and coated and printed, shot peened and coated 316L SS.

Figure 7 shows a comparison between the AP, shot peened, coated and hybrid treated coupons. The peening treatment resulted in a 50% reduction for Ra and 80% for Rz and can be attributed to the fact that as the shots impinge the surface, the rough crests resulting from the printing process are compressed, the protrusions on the surface are deformed radially, forming individual dimples which flatten the surface and thus, reduces the roughness. The surface roughness reduction by SP was also reported by Sugavaneswaran et al. [15] where a 50% deduction in the average surface roughness was discovered after SP AM 316L SS, using S390 shots with a 1 mm diameter, for 15 min, and with a 200% coverage.

### 3.4. XRD Phase Analysis

**Figure 8:**
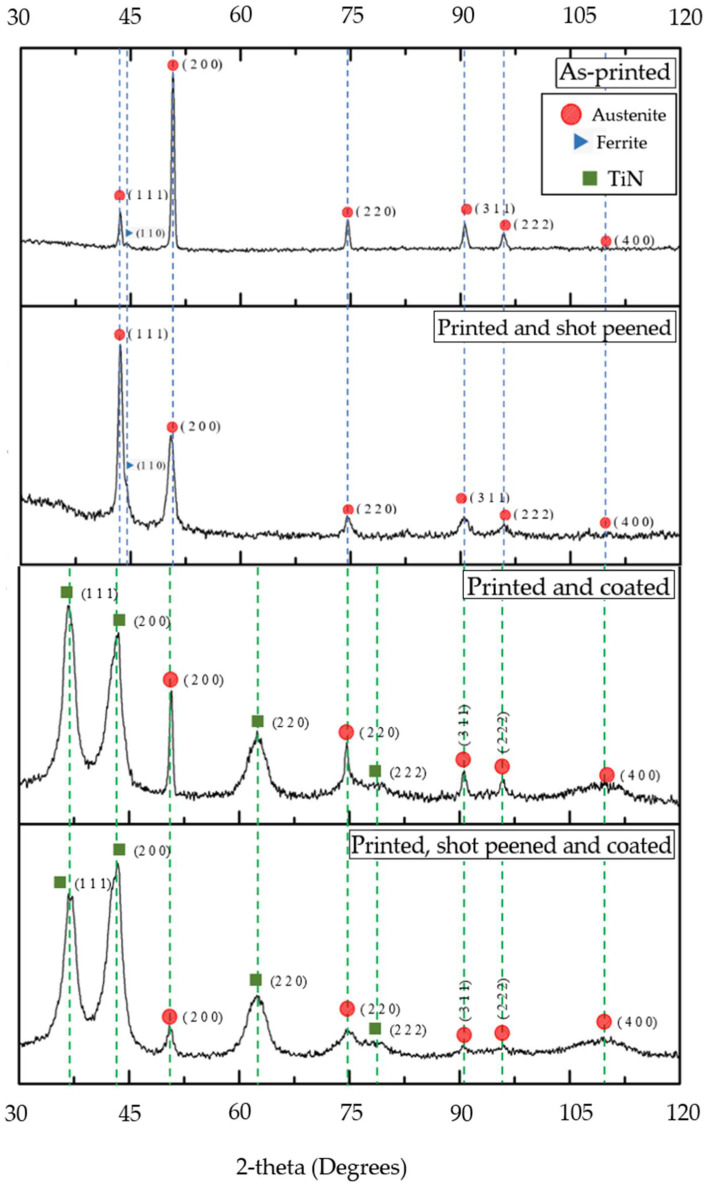
XRD for the as-printed (AP), printed and shot peened (PSP), printed and coated (PC) and printed, shot peened and coated (PSPC).

Figure 8 portrays the XRD diffractographs for the AP, shot peened, coated and hybrid treated coupons. The main differences identified between the AP and shot peened diffractographs were: (i) change in relative intensity at the (111) and (200) peaks, (ii) a poorer definition of the (110) ferrite peak, (iii) broadening of XRD peaks in the shot peened sample and (iv) a slight peak shift for the (200) peak. The broadening and shifting of the XRD peaks are attributed to the macro and micro residual stresses induced by SP [51,52]. Similar differences between the printed and coated, and the hybrid samples were obtained, including: (i) change in relative intensity in the (111) and (200) peaks and (ii) XRD peaks broadening due to the induction of macro and micro-residual stresses.

When analysing the diffractographs of the coated and hybrid treated, the peaks obtained agree with those obtained by Man et al. [53] when studying TiAlN thin films. The two peaks of (111) and (200) for TiN are in the same position as austenite. The XRD technique did not detect any Al and Cu crystalline compounds with the two elements having diffused to form a solid solution. The XRD patterns of the coated surfaces are different from those of the substrate, confirming that there are distinct phases of the coating, even though some of the austenite peaks were still present.

### 3.5. XRD Stress Evaluation

**Figure 9:**
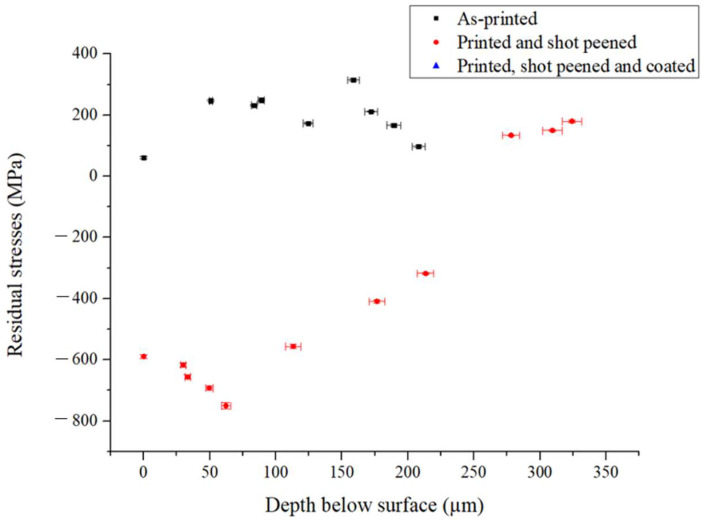
Residual stresses for the AP and PSP against the material depth removed.

A surface residual stress of 61 ± 4 MPa, −589 ± 6 MPa and −693 ± 8 MPa was obtained for the as-printed, printed and shot peened and printed, shot peened and coated respectively. Figure 9 portrays the residual stresses developed along the depth for the as-printed (AP) and shot peened (PSP) samples.

**Figure 10:**
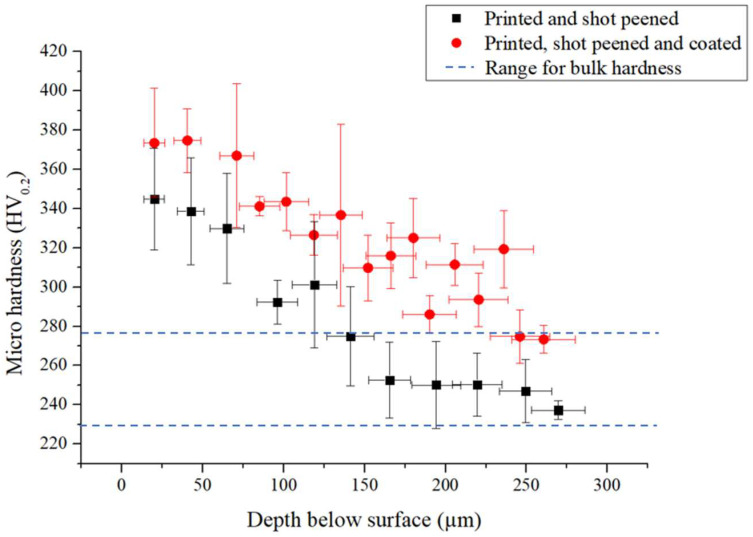
Microhardness-depth profile for printed and shot peened and printed, shot peened and coated 316L SS.

The AP samples exhibited tensile stresses of around 61 MPa, both at the surface and the sub-surface. Tensile stress values have been associated with several mechanisms including: (i) temperature gradient, (ii) re-melting and solidification of layers and (iii) inhomogeneous lattice spacing [54]. On the other hand, the SP treatment induced a maximum compressive residual stress of around 589 MPa. Figure 10 shows that the depth of the shot peened layer is around 250 μm. This is in line with other work on AM 316L SS carried out by Gundgire et al. [16], where the affected depth was in the range of 225 to 275 μm.

Compressive residual stresses for the hybrid treated specimens were similar to those obtained on the shot peened coupon. A surface stress of −693 ± 7 MPa was achieved which reached −561 ± 5 MPa at an affected depth of 54 μm. This outcome shows that the coating deposition on the peened AM specimen did not remove the beneficial compressive stresses induced by the peening process.

### 3.6. Hardness Studies

Table 6 shows that shot peening treatment improves the hardness of the as-printed material by 40%, from 238 HV to 334 HV. This is in line with the study by Gundgire et al. [16], where a hardness of 340 to 360 HV was achieved after SP AM 316L SS. This increase is attributed to plastic deformation taking place during SP. The intrinsic hardness of the coating, which was measured by nanohardness, shows that it further improves the characteristics of the surface of the material. Then, the combination of the shot peening and the coating treatment provides an even superior compound value of hardness, giving 2.9 times increase in hardness over the as-printed samples.

Figure 10 showcases the microhardness depth profile of the shot peened and hybrid sample. It can be noted that the affected depth is also around 250 μm, which is in line with the affected depth obtained in the residual stress measurement (Section 3.5). The affected depth is comparable with that of 189 μm and 225–275 μm obtained by Maamoun et al. [55] and Gundgire et al. [16], respectively.

### 3.7. Material Testing

#### 3.7.1. Adhesion Tests

**Figure 11:**
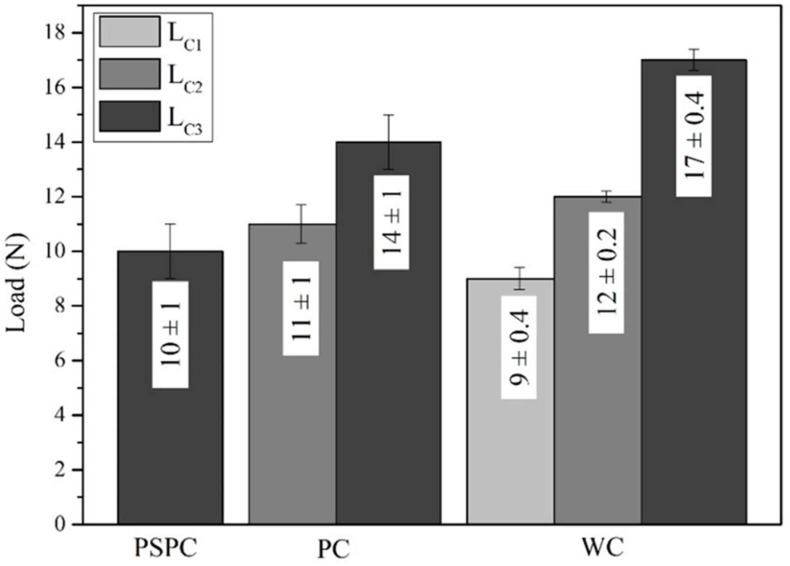
Critical loads achieved at ramped loads for the hybrid, printed and coated and wrought and coated.

The coating and the hybrid treatment showed a similar behaviour of coating characteristics following scratch testing. Figure 11 shows that L_C2_ and L_C3_ were detected along the wear track of coated, while for the hybrid treated L_C3_ only was identified. L_C1_ could not be identified for both variables. The L_C2_ characteristic was made from initial delamination of the coating, while the L_C3_ characteristic was made from interfacial shell-shaped spallation. The earliest sign of adhesive failure and substrate exposure was noted on the hybrid at a distance of 2.6 ± 0.34 mm, and as shown in Figure 11, this corresponds to a scratching load of 10 ± 1 N. This is in contrast with the results obtained in a study by Tillmann et al. [54], in which a CrAlN coating was deposited on AM 316L SS. Both L_C2_ and L_C3_ were obtained at a force of 7 ± 1.7 N and 38.4 ± 3.5 N, respectively. This shows that the CrAlN coating provided better adhesion to the substrate as it failed at higher loads.

**Figure 12:**
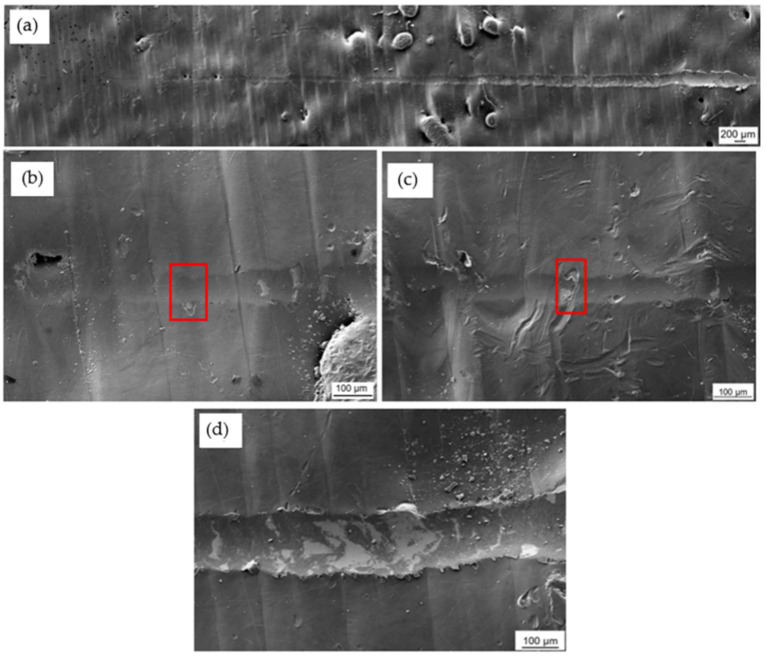
SEM topographical images of the wear scar morphology on the coated specimen: (**a**) Whole length of the wear scar (**b**) Red box showing location of L_C2_ (**c**) Red box showing location of L_C3_ and (**d**) Further delamination along the wear track.

**Figure 13:**
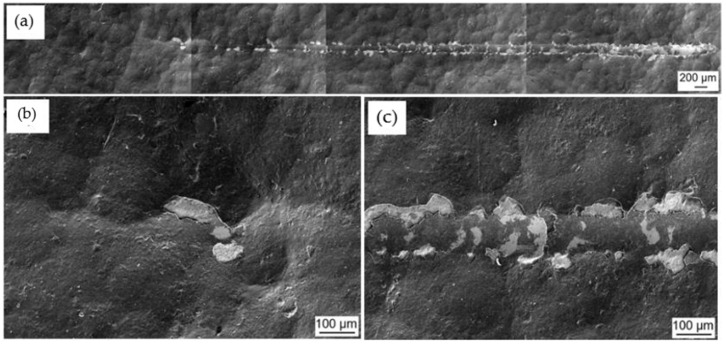
SEM topographical images of the wear scar morphology on the printed and hybrid treated 316L SS: (**a**) Whole length of the wear scar (**b**) Location of L_C3_ and (**c**) Further delamination along the wear track.

Further mixture of cohesive and adhesive failure was identified throughout the wear track of coated and the hybrid treated, showing more delamination and interfacial shell spallation, as shown in Figure 12c and Figure 13c. These were formed as the scratch load increased. Such characteristics were elevated since the soft material was not able to properly support the coating from cracking and forming such defects. As observed in Figure 13c, the perforation of the scar did not result in total coating delamination, even as the load was increased. These were replaced by interval delamination because of residual stress relaxation during coating spallation.

**Figure 14:**
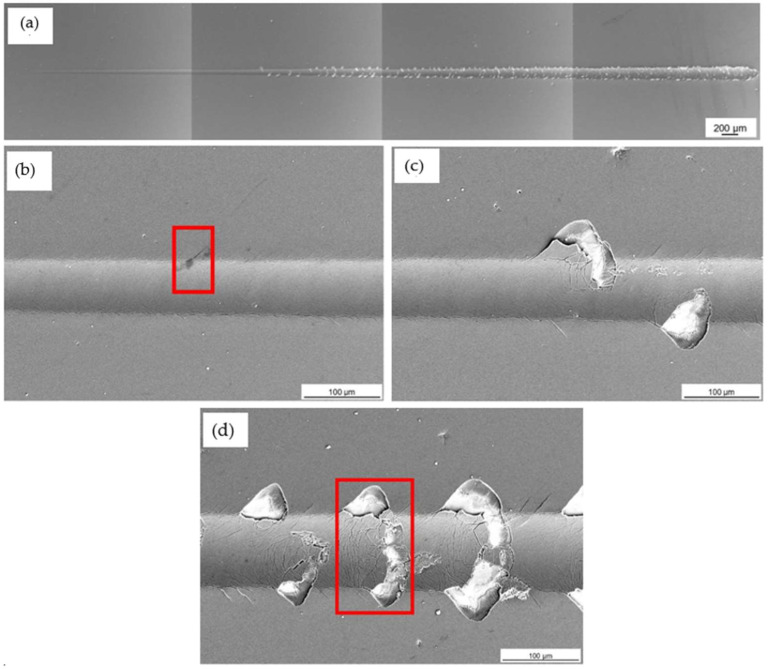
SEM topographical images of the wear scar morphology on the printed coated 316L SS: (**a**) Whole length of the wear scar (**b**) Red box showing location of L_C1_ (**c**) Location of L_C2_ and (**d**) Red box showing location of L_C3_.

Additionally, the scratch testing on the wrought and coated was performed to serve as a control to the coated and the hybrid. During this testing, all the three adhesion characteristics were identified. At low loads, L_C1_ was shown, which was characterised by forward chevron cracks, longitudinal to the scratch track, showing cohesive failure (Figure 14b). Adhesive failure was then identified at a load of 12 ± 0.2 N at which load the coating delaminated along the scratch track (Figure 14c). At further higher loads, L_C3_ (Figure 14d) took place at a load of 17 ± 0.4 N. This characteristic consisted of full interfacial shell spallation, with full delamination of the coating taking place longitudinally along the scratch track. Chevron cracks and localised chipping were found along the track.

When comparing the values in Figure 11, the PC performed better than the PSPC evidenced by the first failure mode detected at a higher load than that measured on the hybrid equivalent. The similar nature of the coated chemical makeup at the surface suggests that the difference in performance can be attributed both to the test mechanics, where the tip interaction changes with the roughness of the sample being measured and the improved load support provided by the harder and stiffer coating. The results of the PC are also superior to the wrought and coated, since the first failure on the wrought and coated was seen at an earlier load. The wrought and coated tests were performed to analyse all the three characteristics synonymous with scratch testing.

#### 3.7.2. Corrosion Tests

##### OCP Curves

The OCP curves for the wrought, as-printed, polished, shot peened and hybrid treated followed a similar behaviour, until they stabilised for the rest of the curve’s duration. This shows that the setup had stabilised and was ready to carry out the cyclic polarisation test. However, the same cannot be said for the coated curve. This is due to the fluctuations in the curve taking place. The reason for these fluctuations could be due to metastable pits growing but their growth is stopped abruptly and repassivated. This repeatable behaviour obtained from a set of repeats for each set, provided an early indication of how the sample was going to perform in the cyclic polarisation test, providing poor corrosion results, performing the worst with respect to the six samples tested.

##### Cyclic Polarisation Curves

**Figure 15:**
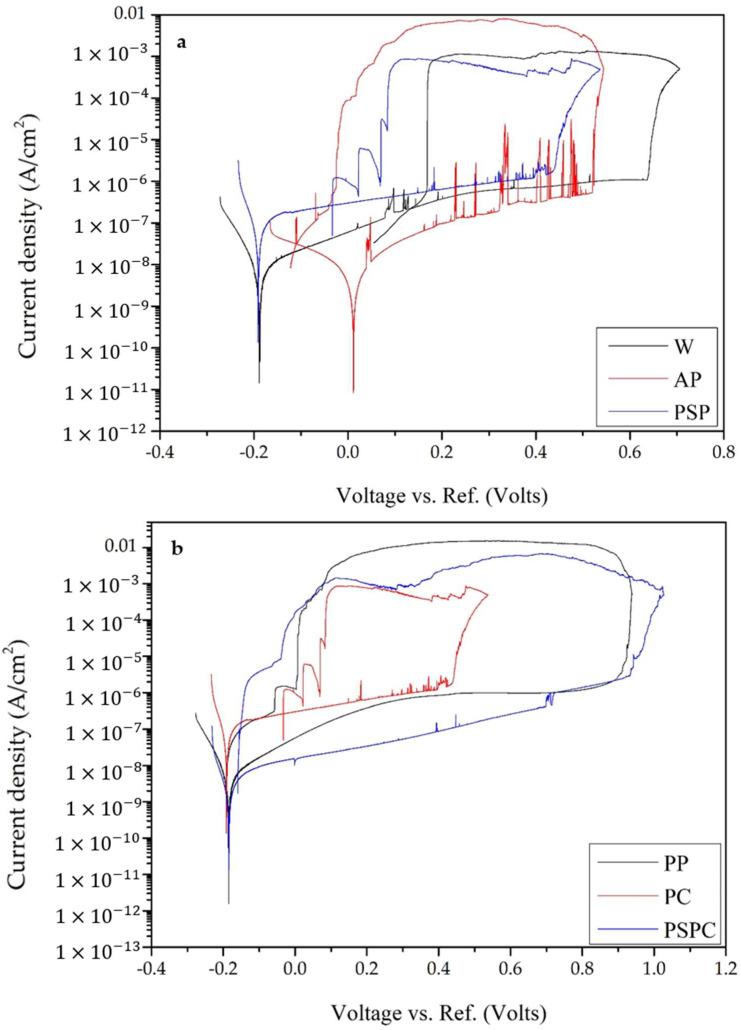
Representative cyclic curves for the six studied surfaces for: (**a**) Wrought, as-printed and shot peened and (**b**) polished, coated and hybrid.

Figure 15 shows the representative curves for the cyclic polarisation tests, while Table 7 provides a summary of important numerical values extracted from the plot and repeats. The most noble E_corr_ was obtained by the AP, whilst the most negative was achieved by the PC sample. The rest of the samples had very similar E_corr_ ranging from −210 to −180 mV. The more noble an E_corr_ is, the lower the corrosion susceptibility [56] and the higher the stability of the passive film [7].

The highest E_break_ was obtained for PP at 776 ± 127 mV, whilst the least was achieved by the PC sample at 241 ± 80 mV. The range for the other samples is between 330 and 690 mV. The E_break_, also known as the pitting potential, is the lowest potential at which the material will succumb to pitting corrosion. Above the E_break_, new pits will form [57]. Therefore, the higher the E_break_, the more resistance to pitting and the further improved stability of the oxide film [56,58].

A fluctuating current density in the passive region of the anodic scans shows the formation of metastable pits [7]. This was showed by the wrought, as-printed, shot peened and coated samples. Their growth is stopped rapidly and repassivated [59]. This repassivation takes place with the aid of the salt films formed by the electrolyte. They suppress the transfer of cations and more growth of the pits. From the curves in Figure 15b, it can be noted that the polished sample showed the lowest metastable pits formation, while the as-printed and the coated showed the most metastable pits formation, with the most fluctuations below E_break_. From this analysis, it can be concluded that the surface roughness impacts the metastable pit formation, the smaller the surface roughness, the less formation of metastable pits. The values of the passive current density go hand in hand with those of the E_break_. The smaller the anodic current, (at E_break_), a denser passive oxide film is formed. Therefore, a smaller current density is preferred. All of the samples had a current density of around 0.4–3 μA/cm^2^, except for the PC, which had a larger current density of around 12 μA/cm^2^, at E_break_.

The E_prot_ is the point of intersection of the forward and reverse scans. The higher the E_prot_, the least prone to corrosion the material is [56]. Therefore, from Table 7, the smallest E_prot_ was identified for the PSPC sample, while the largest was that of the wrought. This shows that the wrought has a stable passive film and pit growth is restricted at an earlier potential value.

All of the six curves show a positive hysteresis loop, which is linked to pitting. The larger the hysteresis loop, the more location for pitting to occur and the less pitting corrosion resistant the material is. Therefore, the bigger the hysteresis loop, the more damage that is occurring on the passive oxide film and the more difficulty to restore it. All the curves show a positive hysteresis loop, with the biggest hysteresis loop identified for the PP. This was determined by finding the difference between E_break_ and E_prot_, as shown in Table 7.

Figure 15a shows the three curves with similar behaviour, that of the W, AP and SP. The results in Figure 15b show that the PC had a poor corrosion resistance when comparing it to the PP and the PSPC, which performed the best. The testing solution damaged the coating and formulated pits which reach the substrate, damaging the sample. As already mentioned, the surface roughness plays an important part in the formation of pits. The PC has a surface roughness of 8 ± 1 µm, which is similar to the 10 ± 3 µm of the AP. This shows that the higher the surface roughness the more surface area for pits to form.

##### Surface Analysis after Testing

**Figure 16:**
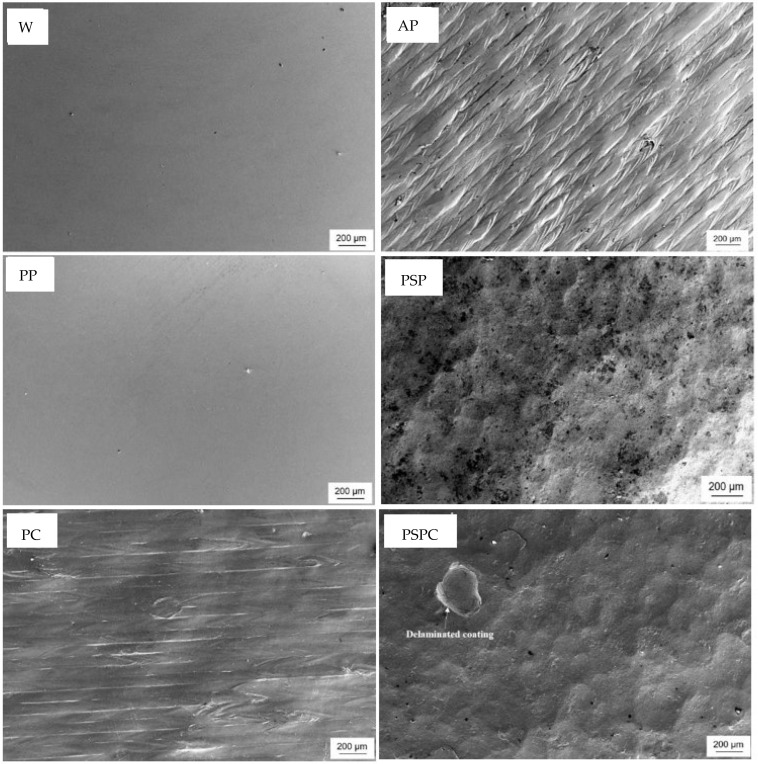
Micrographs of surfaces following corrosion testing.

**Figure 17:**
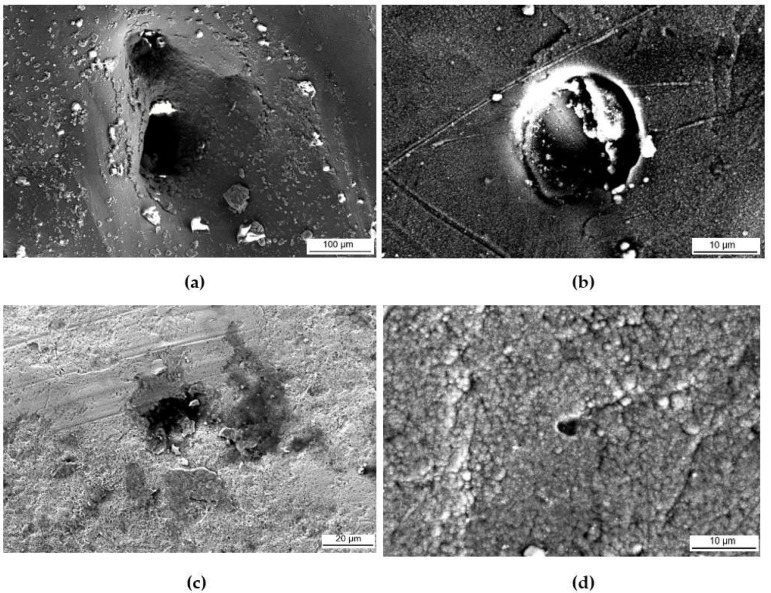
Pits identified on (**a**,**b**) PC, (**c**) PSP and (**d**) PSPC.

Figure 16 shows SEM micrographs of each sample after corrosion testing. Severe damage to the coupons was not evident. However, pits and pores of different sizes were visible on all the samples. In the PC micrographs, the printing striations and directions were revealed, while the PSP and the PSPC samples both show the dimples on the surface which are a characteristic of SP. Additionally, in the PSPC sample, a part of the TiAlCuN coating was delaminated following corrosion testing. Figure 17a,b shows that, upon inspecting the PC sample at a higher magnification, multiple pits were found, as opposed to those found on the shot peened (Figure 17c) and hybrid treated (Figure 17d). On the PSPC less pits were identified, whose benefit will be explained later.

The above demonstrates that the PC has the most corrosion susceptibility, while the PSPC has the least corrosion susceptibility, indicating that the combined effect of the surface treatments of shot peening and PVD provided superior corrosion qualities.

## 4. Conclusions

This study was carried out to analyse the effect of the surface treatments of shot peening and TiAlCuN coating on AP additive manufactured 316L SS, on the surface and sub-surface of the material. The main conclusions from this study include:

- Microscopy and XRD phase analysis showed that the as-printed 316L SS was composed of an austenitic matrix, characterised with columnar and cellular dendritic together, together with the presence of some ferrite.
- XRD stress measurement highlighted tensile residual stresses in the as-printed samples and compressive residual stresses in the shot peened and hybrid treated samples. Compressive residual stresses of 589 MPa for an approximate depth of 250 μm were generated by the cold working achieved by shot peening.
- A 40% increase in surface hardness was obtained on the printed and shot peened specimens, while a 2.9 times increase was achieved following the application of the combined surface treatments. This shows that the coating possesses a high hardness, which when combined with shot peening, improves the material characteristics.
- A 50% decrease for R_a_ and an 80% decrease for R_z_ were found following the application of shot peening on the as-printed specimens. This shows that shot peening has the added advantage of improving the surface finish of additive manufactured components.
- The application of the TiAlCuN coating on the as-printed provided better adhesion characteristics of the additive manufactured 316L stainless steel, than on the hybrid counterpart. This could be attributed to the test mechanics, where the tip interaction is changing with the roughness and the improved load support provided by the harder and stiffer coating.
- The printed and coated combination had the worst corrosion behaviour, while the printed and hybrid treated specimens exhibited the best corrosion behaviour showing that the combined effect of the surface treatments of shot peening and PVD provided optimal corrosion qualities.

The results achieved in this study show optimal qualities for applying a shot peening treatment combined with the deposition of a coating on additive manufactured 316L stainless steel, making this combination of material processing ideal for a range of demanding applications involving bulk mechanical loading, susceptibility to wear under contact loads and corrosion damage, including many such instances found in the maritime transportation industry.

## Figures and Tables

**Table 1:** Main elements of the chemical composition of the wrought 316L SS and the powder used for AM [26,27].

Element	Cr	Ni	Mo	Si	Mn	Fe
(a) 316L SS Powder (wt.%)	17	11	2	1	1	Bal.
(b) Wrought 316L SS (wt.%)	16–18	10–14	2–3	<1	<2	Bal.

**Table 2:** Coating deposition parameters.

Layer Number	Total Time (min)	Time Distribution (min)	Bias Voltage (V)	Nitrogen Flow (%)	Target Current (A)			
					1: Ti	2: Ti	3: Al	4: Cu
Cleaning	10	0	200	0	0.5		/	/
		Ramp up to 2	400					
		8	400					
1: Ti	15	0	90	0	0.5		/	/
		Ramp up to 10			8			
		5						
2: TiN	60	0	90	100	8		/	/
		Ramp up to 30		35				
		30						
3: TiAlN	60	0	90	35	8		2	/
		Ramp up to 5					8	
		55						
4: TiAlCuN	60	60	90	35	8		8	8

**Table 3:** Abbreviations for each sample variable.

Sample	Abbreviation
Wrought 316 LVM SS	W
As-printed 316L SS	AP
Printed and shot peened 316L SS	PSP
Wrought and Coated 316L SS	WC
Printed and coated 316L SS	PC
Printed, shot peened and coated 316L SS	PSPC

**Table 4:** Tensile properties.

	Young’s Modulus (GPa)	Yield Strength (MPa)	Ultimate Tensile Strength (MPa)	Elongation (%)
AP (Measured)	155 ± 7	493 ± 5	644 ± 5	40 ± 2
AP (Literature)	141–183 [33,34]	424–561 [33,35,36,37,38]	528–834 [35,36,37,38]	17–51 [33,35,36,37,38]
Wrought (Literature) [39,40]	193	205–310	515–620	30

**Table 5:** Impact properties.

	Maximum Load (kN)	Total Energy (J)
AM SLM (Measured)	15 ± 0.10	75 ± 2
AM SLM (Literature) [45,46,47,48]	/	60–100
Wrought (Literature) [45,46,47,48]		120–180

**Table 6:** Results obtained from impact testing.

Material	Surface Hardness
AP	238 ± 4 HV_0.2_
PSP	334 ± 16 HV_0.2_
PSPC	691 ± 23 HV_0.2_
TiAlCuN coating *	3022 ± 54 HV

* This measurement was obtained after carrying out a nanohardness test. The other three measurments were obtained via microhardness.

**Table 7:** Average corrosion testing results for each variable.

	Passive Current Density (µA/cm^2^)	E_corr_ (mV)	E_break_ (mV)	E_prot_ (mV)	E_break–Eprot_ (mV)	Presence of Pits and Delamination
W	1 ± 0.1	−197 ± 10	686 ± 24	60 ± 36	626 ± 60	No
AP	0.4 ± 0.2	−2 ± 14	500 ± 36	−133 ± 17	633 ± 53	No
PP	3 ± 0.6	−181 ± 14	776 ± 127	−199 ± 37	975 ± 164	No
PSP	1 ± 0.6	−198 ± 15	334 ± 92	−53 ± 17	387 ± 109	No
PC	12 ± 1	−500 ± 20	241 ± 80	−138 ± 25	379 ± 105	Yes
PSPC	2 ± 0.8	−210 ± 20	595 ± 168	−208 ± 25	803 ± 193	Yes

## Data Availability

Not applicable.
